# Recovery rates, enhanced oil recovery and technological limits

**DOI:** 10.1098/rsta.2012.0320

**Published:** 2014-01-13

**Authors:** Ann Muggeridge, Andrew Cockin, Kevin Webb, Harry Frampton, Ian Collins, Tim Moulds, Peter Salino

**Affiliations:** 1 Department of Earth Science and Engineering, Imperial College, London SW7 2AZ, UK; 2 BP Exploration and Production, Sunbury-on-Thames TW16 7LN, UK; 3 BP Exploration Operating Company Ltd, Aberdeen AB21 7PB, UK

**Keywords:** enhanced oil recovery, crude oil recovery, water injection, miscible gas, water alternating gas, chemical flooding

## Abstract

Enhanced oil recovery (EOR) techniques can significantly extend global oil reserves once oil prices are high enough to make these techniques economic. Given a broad consensus that we have entered a period of supply constraints, operators can at last plan on the assumption that the oil price is likely to remain relatively high. This, coupled with the realization that new giant fields are becoming increasingly difficult to find, is creating the conditions for extensive deployment of EOR. This paper provides a comprehensive overview of the nature, status and prospects for EOR technologies. It explains why the average oil recovery factor worldwide is only between 20% and 40%, describes the factors that contribute to these low recoveries and indicates which of those factors EOR techniques can affect. The paper then summarizes the breadth of EOR processes, the history of their application and their current status. It introduces two new EOR technologies that are beginning to be deployed and which look set to enter mainstream application. Examples of existing EOR projects in the mature oil province of the North Sea are discussed. It concludes by summarizing the future opportunities for the development and deployment of EOR.

## 1. Introduction

The majority of oil companies today are focusing on maximizing the recovery factor (RF) from their oilfields as well as maintaining an economic oil rate. This is because it is becoming increasingly difficult to discover new oilfields. Most of the sedimentary basins that might contain oil have already been explored and new discoveries tend to be small. Those basins that remain unexplored are in remote and environmentally sensitive areas of the world (e.g. the Arctic and the Antarctic). Although there are huge volumes of unconventional hydrocarbons, such as the very viscous oils, oil shales, shale gas and gas hydrates, many of the technologies for exploiting these resources are either very energy intensive (e.g. steam injection into heavy oil), politically or environmentally sensitive (e.g. as seen in recent adverse press coverage of ‘fraccing’ to recover shale gas) or are not yet ready to be applied at scale.

The average RF from mature oilfields around the world is somewhere between 20% and 40% [1–3]. This contrasts with a typical RF from gas fields of between 80% and 90%. At current production rates existing proven oil reserves will last 54 years [4]. This is probably as good as it has ever been. Improving oil recovery to that typical of gas fields could more than double the time for which oil is available or alternatively allow for increased production rates. This would provide more time for an increasingly energy-hungry world to develop alternative energy sources and technologies.

**Figure 1.**
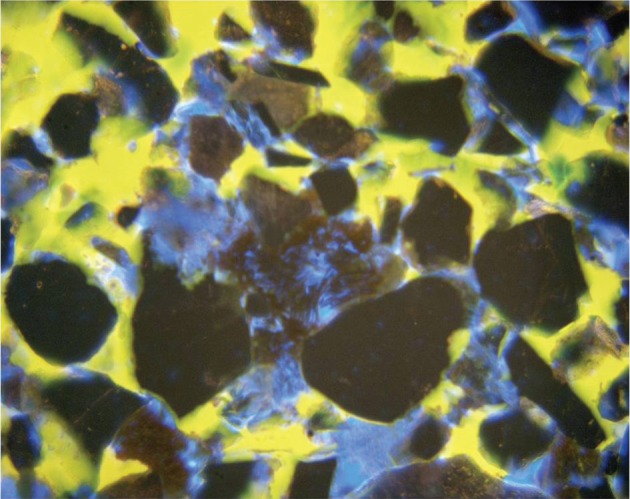
A thin section through an oil-bearing sandstone showing how the oil (dyed blue) and water (dyed yellow) occupy the spaces between the sand grains. The pore space was originally filled with water before oil migrated into the reservoir rock displacing the water.

**Figure 2.**
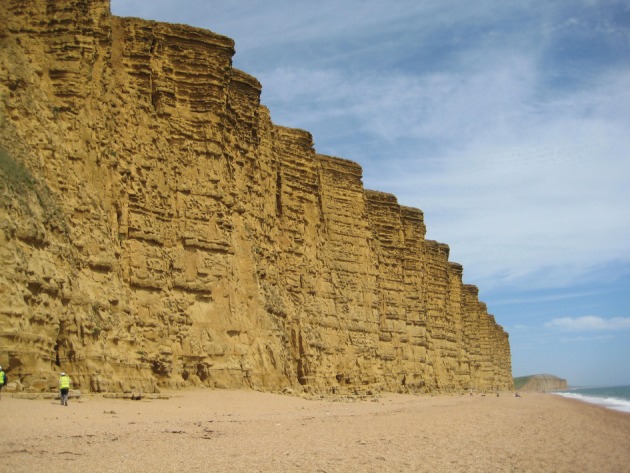
Photograph of the Bridport sands that are exposed in cliffs near West Bay, UK. These rocks form one of the reservoirs in the Wytch Farm oilfield that is found near Bournemouth, UK. (Online version in colour.)

Crude oil and natural gas are found in large underground deposits (usually termed reservoirs or pools) in sedimentary basins around the world. The largest oil reservoir in the world (the Arab D limestone in Ghawar in Saudi Arabia) is approximately 230 km long and 30 km wide and 90 m thick [5]. While most commercially exploited minerals and ores exist as solid rocks and have to be physically dug out of the ground, oil and gas exist as fluids underground. They occupy the connected pore space (figure 1) within strata of sedimentary rocks (figure 2), typically sandstones or carbonates.

Oil and gas are extracted by creating pressure gradients within the reservoir that cause the oil and/or gas to flow through the interconnected pores to one or more production wells. In most oilfields the pressure gradients are maintained by injecting another fluid (usually water but sometimes gas and termed ‘water flooding’ or ‘gas flooding’, respectively) into the reservoir through injection wells. The injected water displaces the oil and occupies the pore space that it originally occupied. By contrast, gas fields are normally exploited simply by reducing the pressure at the production well using compressors. The gas in the reservoir expands as the pressure drops and thus flows to the production well.

Enhanced oil recovery (EOR) involves injecting a fluid into an oil reservoir that increases oil recovery over that which would be achieved from just pressure maintenance by water or gas injection. For lighter oils, these processes include miscible gas injection [6,7], water alternating gas (WAG) injection [8], polymer flooding [9], flow diversion via polymer gels [10] and the use of surfactants [11]. For more viscous (so-called heavy) oils these processes include steam injection and air injection (leading to *in situ* combustion) [12]. The majority of EOR processes used today were first proposed in the early 1970s at a time of relatively high oil prices.

Improved oil recovery (IOR) is a term that is sometimes used synonymously with EOR [13] although it also applies to improvements in oil recovery achieved via better engineering and project management, e.g. identifying volumes of oil that have been bypassed during water injection using seismic surveying and then drilling new wells to access those oil pockets [14,15]. It was first introduced in the late 1980s when the oil price dropped and as a result there was less interest in EOR technologies. At this time there were significant improvements in computer processing speed, computer memory and seismic analysis [16]. Improved computing power enabled engineers to build more complex geological models and thus estimate the effect of reservoir heterogeneity on flow [17–19]. Improved seismic analysis algorithms combined with more powerful computers meant that engineers and geoscientists could use ‘four-dimensional’ seismic surveying, involving the comparison of seismic data taken at different times, in combination with reservoir simulation to identify bypassed volumes of oil on the scale of hundreds of metres horizontally and tens of metres vertically [16]. Developments in measurement while drilling [20] and a new capability to drill deviated, horizontal and multi-lateral wells meant that engineers could target these bypassed accumulations very accurately and drain them.

Using combinations of traditional EOR and IOR technologies it has been possible to achieve RFs of between 50% and 70% [21,22] for some fields but this is still less than the typical RF for a gas field. It is believed that much of this remaining oil is trapped or bypassed in volumes that cannot be accessed by IOR technologies, on lengthscales that cannot be resolved by seismic surveying or accessed by drilling new wells.

New and improved EOR processes are needed to access this remaining oil and improve RFs further while maintaining economic oil production rates. This paper will review existing and emerging EOR technologies, discussing the underlying science, its application and its limitations. In particular, it will focus on recent advances in our understanding of the nature of wettability in rocks and discuss the opportunities arising from the much wider range of polymers that is now available commercially. These two factors have driven a renewed interest in existing EOR technologies and the development of new methods. We will concentrate primarily on the recovery of conventional, light oil from fields that have already been or are about to be developed. The recovery of heavy and unconventional oils will not be discussed in any detail, nor will we consider the recovery of oil by IOR techniques except in passing.

## 2. Why is recovery so low?

Water flooding is currently the preferred recovery technique for most reservoirs because of the higher sustained oil production rates, and the overall higher RFs, that are obtained compared with the case if water were not injected. Oil production without injection is often termed primary recovery. This is because the first wells drilled in a field development are typically production wells to enable oil production and thus the start of income from a field. Where reservoir pressure is well above the bubble point, primary production can be continued for some time before additional pressure support is required to prevent gas coming out of solution in the reservoir.

During depletion, oil flows through the production wells to the surface because the pressure at the base of the well exceeds that exerted by the hydrostatic head of the column of oil in the well. Initially, this occurs naturally but over time the oil rate tends to decrease as the reservoir pressure decreases. In the absence of water injection, pumping may be used to maintain oil rate at economic levels. If reservoir pressure falls below the oil bubble point pressure, gas that was initially dissolved in the oil will come out of solution and, because it has a much lower viscosity, will flow preferentially to the production well. At the same time the viscosity of the remaining oil increases, reducing its mobility further. This will reduce the oil production rate further (although it may increase the total (oil plus gas) production rate through reducing the hydrostatic head in the well). Water (or gas) injection is usually applied before this happens so as to maintain reservoir pressure above the bubble point. For this reason, it is sometimes known as secondary recovery.

Water flooding is relatively cheap, especially for offshore fields because of the ready availability of seawater, although care has to be taken to ensure that the injected water does not result in unwanted, adverse reactions in the reservoir. In some cases injected brines may react with the naturally occurring water in the reservoir (termed connate water) to form scale while injecting very pure water rather than brine may result in clay swelling. Both of these may block the rock pores and reduce the rock permeability. The cost of drilling additional wells for injection is more than outweighed by the increased oil rates that result. Re-injection of gas (produced along with the oil) is used when there is no easy, economic way to export it for sale.

The factors affecting RF from water flooding (and gas flooding) can be understood by considering the following approximate relationship [23]:
2.1
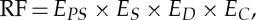
where (i) RF is the recovery factor which is defined as the volume of oil recovered over the volume of oil initially in place (OIIP), both measured at surface conditions. (ii) *E*_*PS*_ is the microscopic displacement efficiency. This describes the fraction of oil displaced from the pores by the injected water, in those pores which are contacted by the water. (iii) *E*_*S*_ is the macroscopic sweep efficiency—the proportion of the connected reservoir volume that is swept by the injected fluid(s). This is principally affected by heterogeneity in rock permeability and by gravitational segregation of the fluids. (iv) *E*_*D*_ is the connected volume factor—the proportion of the total reservoir volume connected to wells. This represents the fact that sealing faults or other low-permeability barriers may result in compartments of oil that are not in pressure communication with the rest of the reservoir. (v) *E*_*C*_ is the economic efficiency factor, representing the physical and commercial constraints on field life such as facilities life, capacity to deal with produced gas and water, reservoir energy (the reservoir pressure may become so low that fluids cannot be produced).

It can even be seen that if each of the efficiency factors is a very respectable 80% then the overall RF is only 41%. Increasing RF therefore requires each of these factors to be increased to close to 100%.

EOR methods are targeted at increasing *E*_*PS*_ and *E*_*S*_ while IOR methods also aim to increase *E*_*D*_ and to some extent *E*_*S*_. Improving *E*_*C*_ is mainly the role of the production and facilities engineers but is also affected by EOR methods if these reduce the amount of water and gas produced alongside the oil, enabling oil to be produced for longer before economic limits are reached.

### (a) Factors influencing microscopic displacement efficiency

The typical microscopic displacement efficiency from a water flood is 70% or less. This is mainly because oil ganglia become trapped in the pore space by capillary effects [24,25] but *E*_*PS*_ is also affected by the relative permeability characteristics of the rock [26], which control the relative mobility of the oil and water when moving through the pore space.

The importance of pore-scale capillary effects on a displacement can be quantified by the capillary number
2.2
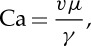
where *v* is the interstitial velocity, *μ* is the fluid viscosity and *γ* is the interfacial tension (IFT) between the displaced and displacing fluid. When Ca<10^−5^ flow is dominated by capillary effects and, in particular, capillary trapping is likely to occur. The typical interstitial velocity in an oilfield displacement (distant from the wells) is approximately 10^−5^ m s^−1^ while the viscosity of a typical light oil is similar to that of water (approx. 10^−3^ Pa s). The IFT between brine and oil is approximately 70 mN m^−1^ so for a typical water flood the capillary number is approximately 2.5×10^−7^. It is generally not possible to apply a sufficiently large pressure gradient between wells to significantly increase the interstitial velocity or to maintain this velocity while injecting a high-viscosity fluid, thus the only way for a reservoir engineer to increase the capillary number is to reduce the IFT. Based on the above calculations this means that the IFT between the oil and the displacing fluid has to be less than approximately 0.1 mN m^−1^ in order to minimize capillary trapping.

**Figure 3.**
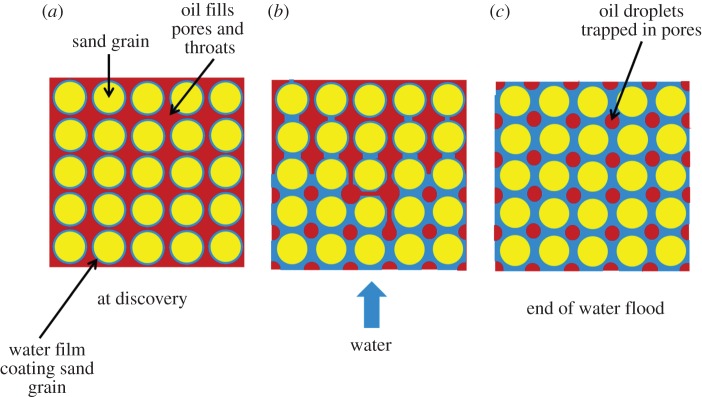
Illustration of oil trapping in a water-wet rock. (*a*) At discovery the sand grains are coated with a thin water film and the pores are filled with oil; (*b*) as water flooding progresses the water films become thicker until (*c*) the water films join and oil continuity is lost.

Both capillary and relative permeability effects are also influenced by the wetting behaviour of the rock in which the oil is found. As discussed in the previous paragraph, if the rock is water wet then there is a higher residual oil saturation (the proportion of oil which remains permanently trapped by capillary effects at the pore scale). This is caused by the growth in the water film on the rock surface during water injection, which ultimately leads to water bridging at the pore throats (so-called snap-off [27]; figure 3) trapping droplets of oil within the pores. As a result, little oil is produced after water breakthrough at the production well unless a higher pressure drop is applied (which is impractical in most cases).

If the rock is oil wet, then the proportion of oil trapped by capillary effects is much lower, as oil continuity is maintained over the rock surfaces and through the pore throats, but water breakthrough is earlier and there is a long period of time during which oil and water are produced simultaneously. The net result is that overall recovery is generally higher [28] if the reservoir rock is oil wet but only after a very large throughput of water.

Most oil reservoir rocks are thought to have a heterogeneous wettability, usually termed ‘mixed wettability’, in that larger pores and throats have both water- and oil-wet surfaces but smaller pores remain mainly water wet [29,30]. It is believed that the reservoir rock changes from an initially water-wet state to this mixed wettability state after the migration of oil into the reservoir [31,32]. Polar compounds in the oil alter the wettability of the rock by a range of interactions including precipitation of asphaltenes, acid–base interactions and ion binding between charged sites on the pore walls and polar hydrocarbon moieties involving higher valency ions in the water that shares the pore space with the oil [31,33–35]. The wettability of reservoir rock thus depends upon its mineralogy, the crude oil composition, the connate water composition and the pore size distribution.

During water flooding in a mixed wettability rock oil and water drain simultaneously through the pore space, snap-off is reduced as most throats have both oil- and water-wet surfaces and thus there is less capillary trapping of oil. This simultaneous drainage of water and oil through the pore space behind the water front combined with the lower residual oil saturation means that more oil is recovered than when the rock is either water or oil wet [36,37].

Increasing microscopic displacement efficiency depends upon finding ways to (i) reduce capillary effects, by reducing the oil–water (or gas) IFT, and (ii) modify the rock wettability to the optimum mixed wettability state.

### (b) Factors influencing macroscopic sweep efficiency

**Figure 4.**
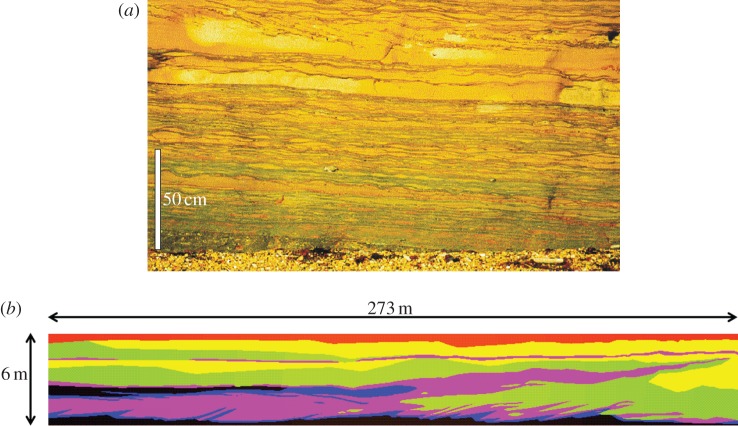
Examples of the types of geological heterogeneities encountered in sandstone oil reservoirs. These examples come from rocks deposited in a deltaic environment. (*a*) Photograph of a heterolithic facies with permeability variations on a centimetre lengthscale vertically and a 10 cm lengthscale horizontally (after Jackson *et al*. [38]). (*b*) Interpreted picture of tidal bar deposits. The lengthscale of these heterogeneities is approximately 100 m. (Online version in colour.)

The macroscopic sweep efficiency of a water flood is principally affected by the geological heterogeneity in the reservoir, which controls the spatial distribution of porosity and permeability. Rock permeability is dependent on the number, size and connectivity of the pores in the rock. The permeability of a typical reservoir rock is approximately 10^−13^ m^2^. A very good reservoir rock might have permeability as high as 10^−11^ m^2^ while a permeability of 10^−15^ m^2^ would be considered very poor. It is controlled by the size of the sediment grains from which the rock was formed, their packing and the subsequent diagenesis (chemical alteration) and cementation (mineral deposition) around those grains. The patterns of grains forming a sedimentary rock depend upon the depositional environment in which the original sediments were formed. These result in permeability heterogeneities with lengthscales from millimetres to kilometres (figure 4).

**Figure 5.**
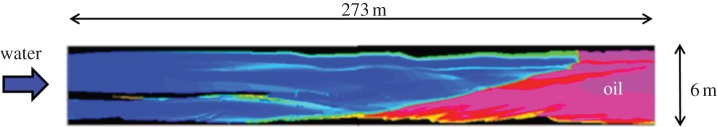
A numerical simulation of a water flood through a heterogeneous reservoir. Flow is from left to right. The oil is coloured red and the water saturation is shown in shades of blue. The water has flowed preferentially through the higher permeability parts of the reservoir, resulting in early water breakthrough at the production well and regions of bypassed oil that will not be recovered.

Higher permeability channels or layers (often described as ‘thief zones’) through the rock are one common, adverse manifestation of geological heterogeneity. The injected water flows preferentially through these zones, bypassing volumes of oil contained in the lower permeability portions of the reservoir. This results in early water production along with the oil and a reduced RF (figure 5).

A particular problem is that the distribution of permeability in a reservoir is usually very uncertain. It is possible to infer the general characteristics of the heterogeneity from the depositional environment and sometimes to correlate specific rock layers between wells, but there is virtually no information about the detailed permeability distribution on smaller lengthscales (e.g. [39,40]). This means that statistical approaches, often based on limited numbers of realizations of the possible reservoir heterogeneity, are needed when attempting to predict reservoir performance (e.g. [19]).

**Figure 6.**
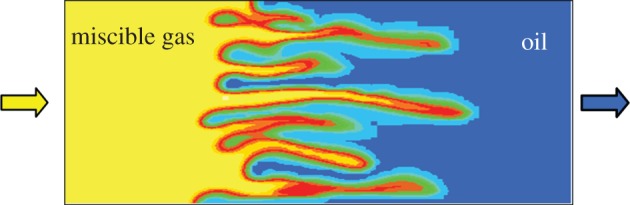
A numerical simulation of viscous fingering seen when low-viscosity gas displaces higher viscosity oil. The viscosity ratio in this simulation is 10. Flow is from left to right. (Online version in colour.)

The effect of geological heterogeneity is exacerbated if the injected fluid has a much lower viscosity than the oil, as is the case when gas is injected instead of water [41–45]. This effect is characterized by the mobility ratio *M*, which compares the mobility of the saturating (*S*) and displacing (*D*) phases in the porous medium
2.3
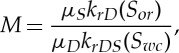
where *k*_*rD*_(*S*_*or*_) is the relative permeability of the porous medium to the displacing phase at the residual oil saturation *S*_*or*_, *k*_*rDS*_(*S*_*wc*_) is the relative permeability of the oil to the displacing phase at the immovable water saturation *S*_*wc*_ and *μ* is the viscosity of the fluid. This is derived from the Darcy equation [46]. The viscosity component of this equation is usually dominant. Even in a homogeneous reservoir, the macroscopic sweep will be reduced when *M*>1 owing to unstable viscous fingering ([43,47–49]; figure 6), where fingers of the displacing fluid develop along the gas–oil interface, rather than the more efficient even contact zone. A typical oil–water viscosity ratio is about 2 while a typical gas–oil viscosity ratio is about 20 [49]. In most cases, it is channelling caused by the reservoir heterogeneity rather than viscous fingering that dominates macroscopic sweep.

Macroscopic sweep may also be affected by gravitational segregation but this is more often observed in gas–oil rather than water–oil displacements because of the higher density contrast between gas and oil [6]. The gas tends to rise above the oil because of its low density and then flow rapidly along the top of the reservoir in an unstable gravity tongue [50] because of its low viscosity. This can result in very early gas breakthrough and poor vertical sweep efficiency.

Improving the macroscopic sweep efficiency depends upon finding techniques that minimize the impact of geological heterogeneity. This is usually achieved by a mixture of viscosity modification of the injected fluid and/or flow diversion in which the water is diverted from the higher permeability zones in the reservoir into the lower permeability rock still containing displaceable oil. In gas floods, it is also important to minimize gravitational segregation.

## 3. Overview of conventional enhanced oil recovery processes

As noted above, the purpose of EOR technologies is to improve the microscopic displacement efficiency and/or the macroscopic sweep efficiency over that obtained from water flooding. Traditionally these involved adding chemicals to the injected water to change its viscosity and/or reduce the IFT with oil, or injecting other fluids into the reservoir (such as carbon dioxide, nitrogen or hydrocarbon gases) that have a very low IFT with the oil (less than 0.1 mN m^−1^). Most EOR processes are thus more expensive to implement than a conventional water flood and only become economically attractive for larger oilfields and when the oil price is high.

### (a) Miscible gas injection

Miscible gas injection is an EOR process that improves microscopic displacement efficiency by reducing or removing the IFT between the oil and the displacing fluid (the miscible gas). When used after a water flood this has the effect of re-establishing a pathway for the remaining oil to flow through and results in a very low residual oil saturation (2% has been measured in reservoir cores recovered from gas swept zones [51]). The drawback of this process is that the gas is both less viscous and less dense than the oil. As a result, these schemes often have a lower macroscopic sweep efficiency as they are adversely affected by viscous fingering [43,47–49], heterogeneity [42,44] and gravity [49,50].

The injected gas may be hydrocarbon gas, carbon dioxide or nitrogen depending on what is available and the reservoir conditions. CO_2_ is miscible with oil at a relatively low pressure and temperature but obviously requires a source of CO_2_. Past applications were in fields near natural sources of CO_2_ [52,53]. It can result in problems with corrosion of steel pipe unless care is taken in the design of wells, flowlines and facilities [54] as well as provision for the separation of the CO_2_ from the hydrocarbon gas when produced. Nitrogen requires a relatively high reservoir pressure for miscibility and involves the use of additional equipment to separate it from the air. As a result, it has not been widely used [53]; the Mexican supergiant Cantarell field is the best known example [55]. Hydrocarbon gas is usually readily available from the field itself or adjacent fields and is thus most widely used, especially in fields where there is no ready market for the gas [22,51,53,56]. In most cases, however, the produced gas that was originally associated with the oil has to be artificially enriched with heavier components in order to make it miscible or nearly miscible with the oil. It may also have to be supplemented with gas from other sources or water injection (see §3*b*) because the volume of produced gas, when re-injected, may not be sufficient to maintain reservoir pressure above the minimum miscibility pressure (MMP).

It is more usual for the injected hydrocarbon gas to be nearly miscible with the oil rather than miscible on first contact. Miscibility then develops between the fluids through the exchange of components, commonly referred to as multi-contact miscibility, resulting in the gas becoming heavier as it passes through the oil and/or the oil becoming lighter [6,7]. However, even if the gas does not achieve full miscibility with the oil there are likely to be pore-scale displacement benefits compared with a water flood as gas components may dissolve in the oil, causing its volume to increase and its viscosity to reduce. As a result it is possible for an immiscible gas flood to result in a lower residual saturation than a water–oil displacement.

### (b) Water alternating gas

WAG injection is an EOR process that was developed to mitigate the technical and economic disadvantages of gas injection. It is the most widely applied and most successful traditional EOR process [8,56].

It involves the injection of slugs of water alternately with gas although sometimes the two fluids are injected simultaneously (termed SWAG). Usually the gas is first contact miscible or multi-contact miscible with the oil but this is not always the case. Injecting water alternately with the gas reduces the volume of gas required to maintain reservoir pressure. It also reduces the tendency for the gas to finger or channel through the oil as the presence of mobile water in the pore space reduces the gas mobility through relative permeability effects [6]. Vertical sweep efficiency is also improved as water, being heavier than oil, tends to slump towards the bottom of the reservoir while the gas, being lighter, rises to the top [8].

Although the majority of WAG applications in the field have been successful, the incremental recovery achieved is generally less than that predicted [8,56]. Early gas breakthrough and a reduced macroscopic sweep, owing to channelling or gravity over-ride, are common. In addition, there are often operational problems. In particular, injectivity can be lower than expected owing to a reduced total fluid mobility near the well as a result of three-phase relative permeability effects and/or a reduced hydrostatic head in the injection well during gas injection.

### (c) Chemical flooding

Chemical flooding is a term that is used to describe the addition of chemicals to the water. Depending on the process, these may change the IFT of water with oil (usually surfactants and alkalis) and/or make the water viscosity match that of the oil (polymers). Chemical flooding has been an option for EOR since the mid-1960s [57]. Early projects using polymer alone were soon supplemented by adding surfactants [58] developed to reduce the water–oil IFT and increase the recovery. Soon afterwards alkalis were added to reduce adsorption of the chemicals by the rock and form added surfactants from charged oil molecules in the reservoir [59].

## 4. Polymer flooding

One means of achieving a more favourable mobility ratio, and thus improve macroscopic sweep, in a water flood is to viscosify the water. This has most often been achieved using high molecular weight water-soluble polymers of 2-propenamide (acrylamide) and 2-propenoic acid (acrylic acid) as the partly neutralized sodium salt in a ratio of about 70:30 of polymer to acid by weight [60,61]. The polymers typically have a molecular weight (or relative molecular mass) of 9–25 million daltons. When dissolved in water, the solutions have a viscosity that depends on the polymer concentration, polymer molecular weight, temperature, water salinity and the concentration of divalent ions. Other polymers, such as xanthan gum [60,61], have been used for the benefit of the improved viscosity yield in more saline water, but these have often been consumed by anaerobic sulfate-reducing bacteria resident in oil reservoirs causing the generation of dissolved hydrogen sulfide (commonly known as known as ‘souring’).

Polymer flooding can recover a substantial increment of the oil in place, typically 8%, at an additional cost of between US$8 and US$16 per incremental barrel [62], but even after 46 years there are difficulties that limit the use of this technology [61]. Large volumes are needed to make the process work at the field scale. The polymers are most effectively supplied as a dried powder but the equipment needed to dissolve them at suitable rates is bulky and there may not be space for this to be retrofitted on offshore platforms. The resultant solutions are vulnerable to shear damage at high shear rates (over about 1000 s^−1^) and are particularly damaged by extensional shear. Increasing the viscosity of the injected fluid inevitably makes it more difficult to inject that fluid into the reservoir and, if the polymer solution has not been properly prepared, debris may actually plug the pore space around the well-bore [63]. Once in the reservoir, the polymer molecules are unstable at temperatures above approximately 70^°^C depending on the water salinity and ionic composition. The mechanisms of thermal degradation are hydrolysis of the amide groups to acid followed by ‘salting out’ (precipitation, mainly driven by the interaction of the acid groups with calcium ions) of the polymer, or free radical (redox) depolymerization resulting in smaller molecules with a lower viscosity.

These difficulties have not prevented the use of polymer flooding in the industry but have limited its extent, with most of the use being in China [64], initially as the result of government policy requiring oil companies to maximize recovery. The recent rise in oil price has initiated a renaissance of the technique among the international oil companies with applications underway in Angola [65] and Oman [66] as well as many being planned for other regions including the UK North Sea [67].

## 5. Alkaline surfactant polymer flooding

Alkaline surfactant polymer (ASP) flooding aims to improve microscopic displacement efficiency by reducing the IFT between the water and oil through the addition of a surfactant to the water, while matching the oil and water mobility through the addition of polymer [68]. Alkali is also added to the water to reduce adsorption of the surfactant onto the pore walls and to control the local salinity to ensure minimum IFT. It can also alter the rock wettability [68–70]. Alkali–surfactant mixtures have also been used to improve macroscopic sweep during WAG. In this process, the gas mobility is further reduced by adding alkali and surfactant to the injected water and thus creating a foam within the pore space [68,71,72]

Like polymer flooding, ASP flooding can significantly improve RFs [73–75] with incremental costs quoted to be as low as $2.42 per incremental barrel for an onshore field [76]; however, like polymer flooding, there are a number of difficulties which have limited widespread field application, especially offshore [61]. Operational difficulties include the large volumes of chemicals that have to be transported to remote sites and then stored on platforms where space is limited. Additional produced fluid processing is needed as ASP flooding results in the production of emulsions with droplets as small as 10 μm in diameter. Finally produced fluids (containing the ASP chemicals) need to be disposed of without impacting the environment. Technical difficulties include the fact that the chemical mix needs to be carefully designed for the fluids to be encountered in the field. ASP flooding works best with relatively low-salinity water (often optimal performance is achieved by the use of a salinity gradient during injection of the different stages), but, offshore, seawater is the only source of injection water so desalination or alternative chemicals may be required.

Despite these difficulties, ASP flooding was applied onshore in the early 1980s when the oil price was high. There has been a recent resurgence of interest as oil prices have increased to the point where the process is once again economic but the only recent large field-scale application outside Daqing [73] in China is in Oman [75].

### (a) Flow diversion

The final type of EOR process commonly applied in light oil reservoirs is that of flow diversion. Unlike other EOR processes, flow diversion does not involve displacing the oil with another fluid, from injection to production well. Instead it involves changing the path of the injected water (or gas) through the reservoir so it contacts and displaces more oil. Until recently, this was typically achieved by injecting a polymer solution with a suitable cross-linker into the higher permeability zones of the formation around the injection well. After injection was complete the polymer and cross-linker reacted to form a gel in the near well-bore region which reduced the absolute permeability of that zone and/or its relative permeability to water [76–80]. The most widely applied treatment of this type consisted of polyacrylamide as the polymer and a Cr(III)–carboxylate complex as the cross-linker [78], although other polymers and cross-linkers are used if the reservoir is particularly hot [81] or the water is saline.

The object of the exercise is to reduce the permeability [78,79] and/or relative permeability [80] of a zone or zones in which water or gas is preferentially flowing. After a successful treatment more water (or gas) will flow into adjacent oil-bearing zones, displacing the oil therein.

Using careful target selection and evaluation and, detailed planning, placement and reagent formulation, many treatments have recovered profit above cost and have been declared commercial successes [79,82,83]. At their best the benefits from such treatments can endure for many years [83], e.g. a well in the Prudhoe Bay field continued producing a high proportion of oil compared with water for 5 years after treatment. In many other cases, however, no benefits were observed or died out after weeks or months [77,83].

**Figure 7.**
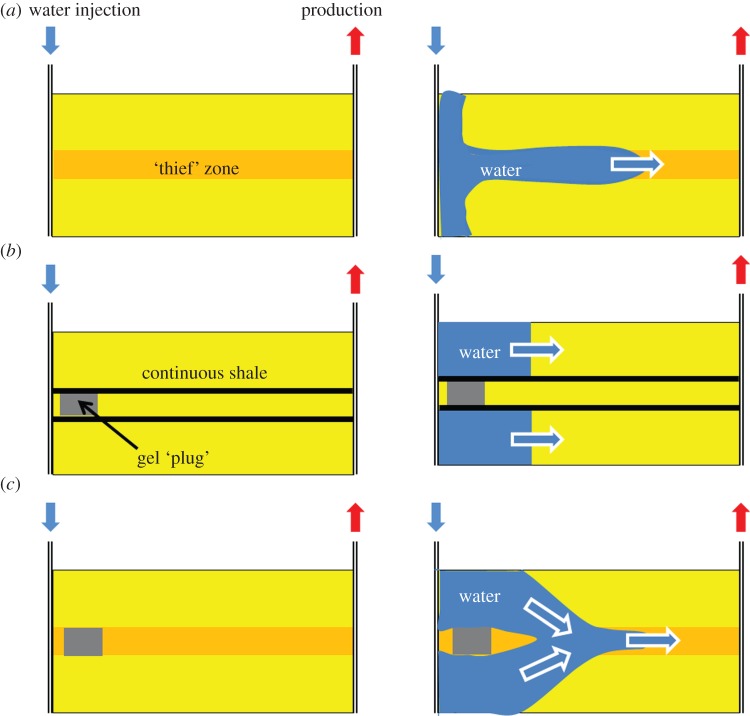
Diagram showing (*a*) how a high-permeability thief zone may result in bypassing of oil in higher permeability zones, (*b*) how a gel plug may successfully divert the water into lower permeability layers if the thief zone has zero permeability shales top and bottom and (*c*) how in the absence of those shales the gel plug will only result in a partial improvement of sweep. The water will flow back into the high-permeability thief zone once the plug has been passed. (Online version in colour.)

In order for this type of flow diversion treatment to be successful, the treated zone needs to be physically isolated from adjacent oil-bearing zones by impermeable shales that extend from the injection well to the production well. If this is not the case then (i) gel may also form in the hydrocarbon-bearing zones, reducing oil production and reducing injectivity/productivity, or (ii) water will simply flow around the zone containing the gel and enter the thief zone further into the reservoir (figure 7) [84]. This is a significant limitation as there are not many reservoirs where the shales are in the right place and laterally extensive, and it is difficult to determine whether such a laterally extensive shale is indeed continuous.

## 6. Enhanced oil recovery deployment

As noted above there are significant practical and economic challenges as well as technical challenges that need to be addressed before EOR technologies can be deployed in the field. Reviewing the development of gas injection as an EOR technology in the North Sea provides insight into the challenges that newer EOR technologies are likely to face in a harsh offshore environment.

We examine a North Sea gas injection EOR scheme that has been in operation for a number of years: the Magnus field in the UK Continental Shelf (UKCS). It is the most northerly producing field in the UK sector of the North Sea. It originally contained approximately 2.4×10^8^ m^3^ (or 1.5×10^9^ barrels) of oil (measured at surface conditions). Like most oilfields, one company operates the field (in this case BP with 85% equity) although it is co-owned by a number of other companies (in this case JX Nippon Exploration & Production (7.5%), Eni (UK) Ltd (5%) and Marubeni North Sea Ltd (2.5%)) [85–88].

The field was initially developed by peripheral water flooding with oil production beginning in 1983. A plateau oil production rate, of 24 000 m^3^ at standard conditions per day (sm^3^ day^−1^), was maintained until 1995, when seawater broke through to wells at the crest of the reservoir. At this stage approximately 40% of the OIIP had been recovered. The remaining oil was believed to be partly trapped on the pore scale (as residual oil) and partly bypassed owing to reservoir heterogeneity with further oil remaining up-dip of the production wells.

Although the residual oil saturation, trapped within the pores, was relatively low at 25% [86], it still presented a favourable target for EOR because of the large volume of OIIP. Surfactant and polymer flooding were ruled out because of the high reservoir temperature (115^°^C). The chemicals existing at that time would have degraded rapidly at these conditions. CO_2_ injection was also deemed infeasible because of the lack of CO_2_ supply and also the costly changes to wells, facilities and pipelines that would have been required to cope with the associated corrosion [55,86]. Nonetheless, the geology of Magnus was felt to create a favourable target for miscible gas injection. The reservoir is formed of repeated layers, in each of which the permeability increases with depth. This increases the tendency of water to sink under gravity towards the bottom of the reservoir, but reduces the tendency of injected gas to segregate upwards, thereby increasing the vertical sweep from the gas.

The injection of hydrocarbon gas was determined to be the best EOR option, as the Magnus oil is sufficiently light and the reservoir pressure sufficiently high for miscibility to be achieved at reservoir conditions with hydrocarbon gas that was relatively lean in heavier components [87,88]. Nonetheless, this option only became reality when suitable gas became available from a number of fields located to the west of the Shetland Islands.

**Figure 8.**
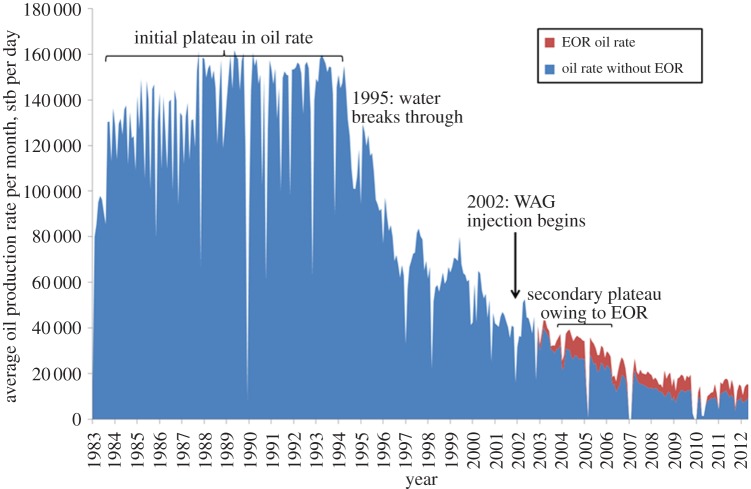
Daily oil production rate (average over a month) from the Magnus field from the start of production in 1983. WAG injection was started in 2002 and by 2005 it was clear that the decline in oil production had been reduced. The oil rate expected without EOR was estimated using numerical simulation. stb, stock tank barrel. (Online version in colour.)

The EOR scheme that was finally implemented in 2002 uses WAG injection. New gas enrichment facilities had to be built at the Sullom Voe Terminal in the Shetland Islands plus over 400 km of new pipeline to transport the gas. The injection rate was maintained as high as possible to limit gravity segregation of the injected gas and water. By 2005, the previous decline in oil rate had been arrested and a secondary plateau in oil production rate was achieved (figure 8). By 2010 some 3.2×10^9^ sm^3^ of gas had been injected into four gas injection wells yielding 1.8×10^6^ sm^3^ of incremental oil overall and contributing 40% of the oil production rate in 2010 [88].

Delivering this additional oil required significant changes to both the operation of the field and the way its performance was monitored. This is because operating an EOR WAG injection scheme is inherently more complex than operating a primary recovery process or water injection scheme. Four aspects of this additional operational complexity are described below.

### (a) Water alternating gas changeovers

The aim was to operate two wells on gas injection and two wells on water injection at any given time, swapping them between water and gas injection at appropriate intervals. It was intended that equal reservoir volumes of water and gas would be injected during each cycle. The operation to change each well over from water to gas injection, and vice versa, takes 3 days and involves physically removing the injection line for one phase and replacing it with the injection line for the other phase. These changeovers need to be scheduled in advance to fit in with other planned platform activities. Unfortunately, they are perceived by the platform staff as low priority with little associated cost in delaying them, although in reality delaying changeover reduces the benefit of the WAG process by changing the effective WAG ratio. This is illustrated by a combination of events that occurred in late 2008 that resulted in one of the gas injection wells being temporarily shut-in. This caused a change to the effective WAG ratio in the remaining gas injection well. This in turn resulted in more gas being produced, because the gas mobility was not being reduced by a following slug of injected water, to the extent that the ability of the platform to manage the volume of returned gas was exceeded. Consequently, production from all the EOR production wells was cut back, reducing the oil production rate.

### (b) Measurement of gas flow rate

Accurate measurement of gas volumes over time, and allocation to the various injection and production wells, is important to maintain efficient WAG scheduling and thus the overall efficiency of the EOR process. This meant that all wells that inject gas should have individual flow meters. These would not be required for an ordinary water flood. Even retro-fitting flow meters to these wells was challenging as it competed for offshore time against another major project to construct additional drilling slots, and later against drilling activity.

### (c) Reservoir pressure management

Average reservoir pressure in those parts of the reservoir subject to the WAG scheme has to be maintained above the MMP of 34.5×10^6^ Pa. However, this is also the pressure rating for the EOR production wellheads, which in turn places an upper limit on the reservoir pressure. After allowing for safety margins and worst case conditions in which the production tubing would be full of gas, this combination of MMP target and wellhead pressure rating created a narrow range for the reservoir pressure. As a result, the reservoir pressure must be routinely measured in those parts of the reservoir subject to WAG, which would not be done for an ordinary water flood.

### (d) Gas supply

The injected gas is imported from other producing oilfields. Operational issues at these other fields have led to a variable gas supply at Magnus, resulting in a less efficient sweep through the reservoir.

## 7. Emerging enhanced oil recovery technologies

The traditional EOR technologies (miscible gas/WAG and chemical flooding) for improving microscopic displacement and macroscopic sweep have been around for a long time but significant technical, operational and economic difficulties (such as discussed above) continue to limit their application and the volume of oil recovered when they are implemented at scale.

Several completely new technologies have been developed over recent years that aim to improve recovery using rather different mechanisms from those used by the traditional EOR techniques. They benefit from significantly lower cost per incremental barrel, have broader applicability, are less dependent on detailed characterization of the reservoir rock and fluids and are less complex to implement.

In this section, we examine two of these new processes (low-salinity water flooding and deep reservoir flow diversion), as well as considering the time taken from identification of the process in the laboratory to implementation in the field.

### (a) Low-salinity water injection

Low-salinity water injection is a recently developed EOR process that improves microscopic displacement efficiency by modifying the reservoir wettability. As noted above, most oil reservoir rocks have a heterogeneous or ‘mixed’ wettability. The effect of the low-salinity water is to make these rocks slightly more (but not completely) water wet as it progresses through the reservoir. This has the effect of mobilizing more of the oil behind the displacement front and increasing recovery.

The potential to use wettability alteration as the main recovery mechanism in an EOR process has only recently become a major topic of research. This is despite the fact that it was first recognized in 1959 by Wagner & Leach [69] and tested in the field in 1962 [89]. These workers controlled the wettability through adjusting the pH and sodium content of the injected water. Wettability alteration was recognized as a secondary recovery mechanism in ASP floods (resulting from the addition of the alkali to the chemical mix in the injected water [68]) but developments of these floods focused primarily on minimizing the IFT.

The recovery process involves injecting brine, with a low salinity and that is depleted in divalent cations (compared with the *in situ* brine), into sandstone reservoirs. It should be noted that the salinity should be as low as possible without adversely affecting flow performance. It does not usually involve injecting pure water as this can reduce oil recovery by causing swelling and deflocculation of some types of clay minerals and subsequent blockage of the pore space. After the first laboratory investigations of the effect of water composition on oil recovery in 1959 [65] and 1967 [90], no further systematic laboratory studies of the effect were performed until the 1990s [91–94]. These and other studies performed in BP (1997–2002, unpublished data) resulted in the first field test in a single well in 2004 [95]. Several other such tests [96] led eventually to an inter-well field trial in 2010 [97]. All confirmed that lowering brine salinity increased oil recovery.

All the core flood and field evidence is consistent with the theory that low-salinity water injection progressively modifies the reservoir wettability through multi-component ion exchange [98,99]. As noted in [33], one of the mechanisms causing areas of the pore walls to be oil wet is ion binding between the oil and mineral surface, mediated through multi-valent cations such as Ca^2+^, Mg^2+^ and Fe^2+^. Injecting lower salinity water that has a reduced concentration of these divalent cations results in this ion-bound oil being released from the mineral surface (usually, but not exclusively, kaolinite) and that part of the surface becoming water wet. It is important to note that this only has a slight effect on the bulk rock wettability—overall it still has a mixed wettability. It is just slightly more water wet. Recent academic studies are providing support for the proposed mechanism [100–103].

Seventeen years after the publication of initial investigations into the impact of water composition on oil recovery by Jadhunandan & Morrow [92], EOR by low-salinity water injection is about to be deployed in the second phase of the Clair field development, Clair Ridge, in the UKCS. Reservoir condition core flood tests using Clair rock and oil showed significant benefits to secondary low-salinity flooding with a reduction in residual oil saturation of between 5.6% and 7.6% [104].

This long time frame to take preliminary research to deployment is typical. It was a function of the need to confirm the results under realistic reservoir conditions using real reservoir rocks and fluids, and also to develop a sufficient mechanistic understanding to convince business managers and partner companies that the technology was robust. Further time was taken to scale up the technology to field scale including identification of suitable desalination plants that could be operated safely in harsh offshore conditions.

### (b) Deep reservoir flow diversion

Deep reservoir flow diversion is a recently developed EOR technique for improving macroscopic sweep efficiency. It was recognized that, while flow diversion by polymer gels in the reservoir adjacent to the injection well could be very successful [79,82,83], in many cases cross-flow in the high-pressure gradient environment near the well meant that the diverted water soon flowed back into the thief zone [84]. It was then realized that chemical treatments placed deep in the reservoir would not be so vulnerable to this, but rather would benefit from the diversion of fluid in the interval between the injection well and the reduced permeability zone [105].

The challenge was to identify a chemical that would only reduce the permeability of the thief zone when it reached the right part of the reservoir. An early attempt involved using a water-soluble polymer mixed with hydrolysed aluminium citrate as the cross-linker [103]. It was believed that the cross-linker and polymer would travel together through the reservoir and form a permeability-reducing gel phase on heating, but this proved unreliable, probably because of chromatographic separation of the polymer and cross-linker and/or precipitation of the metal ion [106]. The lesson from this field trial was that the blocking agent had to travel through a large amount of rock and that it should be a single component to prevent deactivation. It was concluded that it should be particulate, inert and compact when travelling through the rock pores to the target location, then when triggered should expand and block the rock pores. The trigger selected was the temperature difference between the injected water (which coming from the surface is initially cooler than the reservoir) and the reservoir. Over prolonged injection, cooled zones are created around the injection well resulting in a thermal gradient between injection and production wells.

A particle system was developed consisting of water-soluble polymer backbones linked together with permanent cross-linker in sufficient quantity to allow them to swell significantly in water. A larger amount of thermally breakable cross-linker was added to lock the particles into their manufactured particle size. This was achieved by polymerizing the monomers as an emulsion in light mineral oil [107,108].

To deploy this system, a surfactant is added to the injection water followed immediately afterwards by the particle dispersion. The natural turbulence in the well-bore is sufficient to cause the oil in the formulation to emulsify and the particles to be individually wetted by water. The dispersion of particles in water continues down the injection well, into and through the pores of the formation rock. The water-particle system travelling down the thief layers is progressively heated by the unswept layers above and below (which do not contain injected water and are thus at the original reservoir temperature). Eventually the water–particle system is heated to the point where the temperature-sensitive cross-links are broken and the particles absorb the water, swell and block the rock pores. The permeability of the rock in the thief zones is reduced and the subsequent water injection is diverted into the oil-bearing lower permeability zones to displace oil towards a producing well.

The technical field trial of the ‘temperature-triggered’ particles took place in 2001 [109,110] followed by commercial field trials in 2004 through to 2007 [111–113]. Following successful incremental oil production results, the technology deployment started in 2007. The first 19 treatments produced over 200 000 m^3^ of incremental oil [114]. To date approximately 80 treatments have been completed with significant incremental oil recovery over the water flood and a success rate in excess of 80%. Pressure fall-off tests suggest that the blockages have been formed in excess of 100 m into the reservoir, which is desirable for maximum flow diversion with minimum decrease in water injectivity [111].

Other treatments have been proposed based on intra-molecularly cross-linked polymer- [115], salinity- [116] or pH-triggered systems [117] or pre-formed gel particles [118–120]. It is believed for various reasons, though not universally accepted, that the intra-molecularly cross-linked polymer systems do not propagate as far into the reservoir pore systems as the temperature-triggered particles (e.g. [120] and the references therein).

Again it is interesting to note that it took more than 15 years for this technology to be deployed after its initial conception leading to publication in 1992 [105].

## 8. Discussion and conclusion

Crude oil is expected to supply 20–25% of the world's energy by 2035 [121]. Most of this is expected to come from conventional crude oil [3] and mature fields. This suggests that there will be an increasing application of EOR in order to increase the RF and oil production rate from these fields.

**Figure 9.**
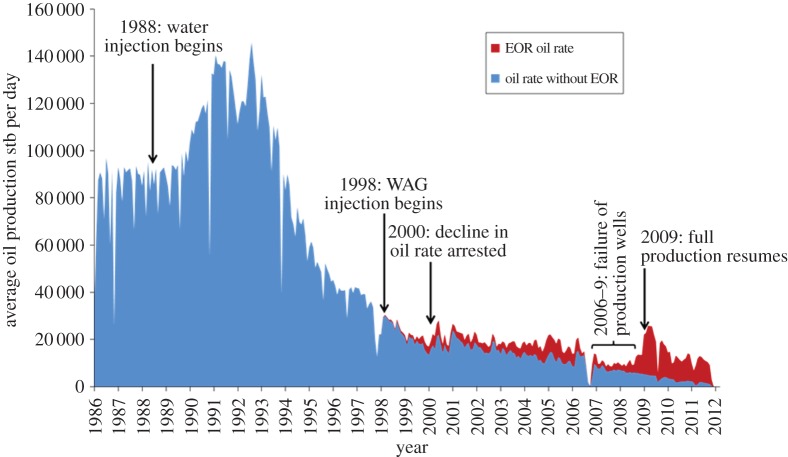
Daily oil production rate (average over a month) from the Ula field from start of production in 1983. WAG injection was started in 1998 and by 2000 it was clear that the decline in oil production had been stopped. Today almost all the oil production is believed to have come from EOR. The oil rate expected without EOR was estimated using numerical simulation. (Online version in colour.)

We have seen that traditional EOR technologies can be very effective at improving recovery, especially through increasing microscopic displacement efficiency (table 1). When they are implemented, they can be very successful, e.g. as in Magnus (figure 8) and in the Ula field in the North Sea where almost all the oil produced is believed to come from WAG injection (figure 9). They are, however, often complex to design, develop and operate, as we have seen through the summary of WAG deployment in the Magnus field. Furthermore, in many mature fields offshore (such as in the UKCS) it is impossible to implement EOR because of the lack of space on the platforms for the additional equipment needed to inject different fluids and/or process the produced fluids. The response to the application of these techniques, in terms of increased oil production rate, is usually slow, typically months or years after the process is initiated. These issues, combined with the use of large quantities of expensive chemicals or valuable hydrocarbon gases, means that they are only economical when the oil price is high.

**Table 1.** Comparison of EOR processes with water flooding in terms of their microscopic displacement efficiency and macroscopic sweep efficiency, together with a summary of their limitations.

	microscopic displacement efficiency, *E*_*ps*_	macroscopic sweep efficiency, *E*_*s*_	
EOR process	compared with water injection		limitations
miscible gas injection	+	−	very sensitive to heterogeneity
			poor vertical sweep owing to large density difference from water
			reservoir pressure must be greater than minimum miscibility pressure
			excess gas production
WAG injection	+	+	operationally more complex
			oil may be trapped in pores by water if too much water injected
polymer flooding	∼	+	well injectivity owing to higher viscosity of injected water
			loss of polymer by adsorption
			cost due to large volumes of chemical required
			may not be feasible in hot reservoirs or those with saline water
ASP flooding	+	+	complex to design, requiring analysis of oil, water and rock chemistry as well as geological heterogeneity
			cost due to large volumes of chemicals required
			may not be feasible in hot reservoirs, carbonate reservoirs or those with saline water
low-salinity water injection	+	∼	mechanism not fully understood
			possible dilution of injected low-salinity water by *in situ* brine
polymer gel treatments at injection wells	∼	+	only works where high-permeability thief zone is isolated from other oil-bearing zones
			may not be feasible in hot reservoirs, carbonate reservoirs or those with saline water
			potential production of H_2_S by sulfate-reducing bacteria in reservoir
deep reservoir flow diversion	∼	+	only works for water injection
			may not be feasible in hot reservoirs, carbonate reservoirs or those with saline water

Traditional miscible gas EOR techniques are very sensitive to geological heterogeneity and so additional work must be performed to evaluate the reservoir description before development proceeds. Chemical flooding techniques also require a good understanding of the chemical behaviour of the rock and may need careful selection of chemical(s) that are robust to the temperature conditions in the reservoir.

New EOR technologies are needed that are easier to design, require less specialist equipment and produce a quicker response in terms of oil rate. This is particularly the case for mature off-shore fields where there is little space for additional equipment on platforms. This also suggests that companies should be planning the deployment of both new and existing EOR technologies at the beginning of field development to ensure that there will be facilities and space to implement EOR in due course. In many cases, maximum oil recovery is only achieved if EOR is deployed as soon as production begins. The traditional approach of only moving to EOR after oil rates from a water flood drop results in significant volumes of oil being bypassed.

A major challenge remains the time delay between the deployment of a given EOR process in a field, often involving considerable extra capital and operational costs, and the response in terms of additional oil production. The benefits from drilling additional water injection wells are usually seen within months while it may take a year or more before incremental oil resulting from an EOR scheme reaches the production wells.

We have discussed two emerging EOR technologies (low-salinity water injection and deep reservoir flow diversion) that remedy some of the drawbacks of traditional EOR processes. Low-salinity water injection is simple to plan and deploy as it is very similar to conventional water flooding. There are no additional chemicals required or additional processing after production. The main additional cost (and limiting factor on existing platforms) is the need for desalination equipment. Deep reservoir flow diversion has proved to be more successful than traditional near-well gel treatments because it is less sensitive to the nature of the geological heterogeneity in the reservoir. It therefore requires less reservoir characterization and few reservoir simulation studies before implementation. In addition, it does not require any upfront capital investment.

It is probable that there will be further developments in enhancing water flooding. We have described how low-salinity water injection improves oil recovery in sandstone reservoirs by making the rock more water wet. A similar change in wettability has been observed in chalk when seawater enriched in Ca^2+^, Mg^2+^ and SO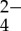 is injected [122], although other workers suggest that viscosity alteration and formation of a microemulsion between oil and water may also improve oil recovery [123].

Further developments are probable in EOR technologies that improve macroscopic sweep. The deep reservoir flow diversion technique described above is designed for water flooding. Similar technologies are required for gas flooding, especially if CO_2_ injection for EOR and geological sequestration of the CO_2_ is to succeed.

Increasing or even maintaining crude oil production to help supply the world's energy demand is likely to adversely affect climate change unless it is associated with geological carbon sequestration. Oil reservoirs are ideal candidates for secure storage of anthropogenic CO_2_ because they are known to have trapped oil for millions of years. CO_2_ injection is also able to significantly improve oil recovery [124], although in this case there is still a net increase in CO_2_ emissions, i.e. more CO_2_ is produced from burning the additional oil than is stored in the reservoir by injection [125]. To achieve this will require the development of technologies to improve the macroscopic sweep efficiency of this process in order to maximize the trapping of CO_2_ in the reservoir while maximizing oil recovery and production rate. It will also require additional political and financial support to put in place the carbon capture facilities at power stations, the pipelines for distributing the CO_2_ to the oilfields and new pipelines and facilities that are resistant to corrosion in existing oilfields [126].

Another EOR technology that we have not discussed in any detail is that of microbial EOR. This uses native or introduced microbes to improve oil recovery via a variety of mechanisms including flow diversion, *in situ* upgrading, wettability modification and generation of biosurfactants within the reservoir [127]. Although it was first proposed by Zobell [128] in 1947 and has been the subject of much research since then [127–130], it has not been widely applied. This is probably because it has proved to be difficult to predict performance in the field. With recent advances in the biological sciences and the modelling of biological processes, it is possible that microbial EOR or methanogenesis [129] may yet be more widely applied in the future.

EOR projects are going to become increasingly common worldwide in the future, despite concerns about greenhouse gas emissions, as demand for oil will continue to increase [3] while at the same time it becomes harder to find new oilfields. We have not yet achieved the technological limit in terms of the RF that can be obtained using these processes. At present, their deployment is controlled by economic factors and operational constraints. Research continues to try and mitigate these factors and constraints, as well as to develop more advanced and effective recovery processes, but the challenge in all cases is to move these technologies more rapidly from the laboratory to the field.
